# Building games into multicenter clinical trial systems to boost trial engagement

**DOI:** 10.1186/s13063-026-09498-6

**Published:** 2026-02-13

**Authors:** Ryan Majkowski, Shannon Hillery, Bradley J. Barney, Paul Ryu, Esther Woo, Nichol McBee, Andrew Mould, Lindsay M. Eyzaguirre, Elizabeth Holthouse, Karen Lane

**Affiliations:** 1 Department of Neurology, Trial Innovation Center, BIOS Clinical Trial Coordinating Center, Johns Hopkins University School of Medicine, Baltimore, MD USA; 2 University of Utah Health, Salt Lake City, UT USA

**Keywords:** Workplace gamification, Clinical trial performance, Site start-up, Multicenter clinical trials, Trial Innovation Network

## Abstract

**Background:**

Workplace gamification refers to the translation of ordinary work tasks into a fun thematic framework—using game design elements such as points, competition, and recognition—to enhance engagement and motivation. Clinical trials are lengthy and operationally demanding, leading to low enthusiasm and disengagement. As a trial coordinating center, we successfully put gamification to work as an engagement tool in a series of multicenter clinical trials.

**Methods:**

We combined trial metadata with gamification and concepts around motivation to enhance how we engaged site teams responsible for trial activation, patient recruitment and retention, protocol compliance, and data quality. Using metrics routinely captured within trial data platforms, performance indicators were extracted and converted into point-based scoring systems. Gamified strategies were integrated into start-up and enrollment phases of each clinical trial, as both phases had measurable tasks with defined timelines.

**Results:**

We successfully gamified the start-up tasks of seven trials and enrollment tasks of nine trials. As start-up tasks were similar, one game fits multiple trials. Gamification was customized, however, for enrollment processes and further adapted to address protocol-specific challenges and periods of suboptimal performance. Drawing from these experiences, we present a set of guidelines that outline key principles and gamification mechanics, serving as instructions for game development in this context.

**Conclusion:**

Gamification guidelines afford a novel approach to align intrinsic motivators for achievement with existing trial infrastructure and performance metrics to enhance trial team engagement across diverse trial settings.

**Supplementary Information:**

The online version contains supplementary material available at 10.1186/s13063-026-09498-6.

## Background

Clinical trial site teams take on multiple, often competing responsibilities: delivering patient care, advancing knowledge through teaching, and integrating clinical trials into routine practice. With so many projects and novel treatments to learn and document, site teams often have difficulty allocating time for important trial tasks such as reviewing a new protocol training module or coordinating submissions to their Human Research Protection Program (HRPP) representatives. A 2024 survey involving over 850 global site staff found that 70% reported that trials have become substantially more difficult to manage due to escalating complexity, resource constraints, and burdensome technologies [1]. These challenges are magnified by isolation, as site teams operate remotely from coordinating center personnel, sponsors, and other site teams. This separation often leads to uncertainty regarding priorities and decision-making.

How do we help site teams not lose sight of a trial timeline, make trials more approachable, and create an atmosphere where goals feel more attainable? To be attainable, goals must not only be within reach but also rewarding and valued [2, 3]. There must be an urgency felt by the site team. Because the distinctions between extrinsic and intrinsic motivation function similarly in both work and play, gamification may be an effective way to amplify engagement and help site teams achieve trial goals. Gamifying site tasks can highlight the needs of a trial and provide both intrinsic and extrinsic motivators to investigators and their teams through game play, awards, and competition. Added engagement, in turn, could make a significant contribution to multicenter clinical trial productivity [4]. Our previous research suggests a relationship between game enjoyment and faster task completion during trial start-up [5]. This former work validates gamification as a potential method of improving trial site performance.

There are five important intrinsic motivators: Autonomy (I control), mastery (I improve), purpose (I make a difference), progress (I achieve), and social interaction (I connect with others) [6, 7]. Gamification has the potential to increase intrinsic motivation by providing site teams with experiences that satisfy these five universal psychological needs and serve as alternative strategies to improving productivity, despite what might appear to be the absence of explicit trial-related practical purposes. Gamification can serve as a method of recognizing the often-overlooked day-to-day tasks that are fundamental to overall trial success. Recognizing which tasks are important to study leadership can signal that the work is meaningful, further increasing intrinsic motivation [8, 9].

Extrinsic motivation may be easier to create than intrinsic motivation, as tangible rewards given at specific time points are easier to organize than tapping into what psychological effects drive individual team members and interest them in gameplay. Perryer et al. [3] aptly describe extrinsic motivators as effective only when the “desired outcome has been achieved.” In the clinical trial space, extrinsic motivators such as financial incentives have been effective in the start-up phase [10], but this represents a short-term tangible goal, not a solution to ongoing challenges. The short-term effects of such methods on motivation are unlikely to be sustained, as enthusiasm for game features, in and of themselves, seems to decrease over time [4]. Therefore, extrinsic motivators like financial incentives and points should serve as a method to emphasize intrinsic value when gamifying tasks [3]. Most trials are lengthy and operationally demanding, and gamifying trial activities has the potential to sustainably engage site teams and boost motivation. Recently, we reported that when gamification engaged site teams with public recognition and awards, site teams who reported having enjoyed the game met start-up timelines more often [5]. Otherwise, the literature supporting the use of games in the clinical trial workplace is sparse. Our Trial Innovation Center (TIC) team, supported previously by the National Institutes of Health and National Institute of Neurological Disorders and Stroke and currently by the National Center for Advancing Translational Sciences (NCATS) and the National Institute on Aging (NIA), has been developing games since 2012 to boost engagement during various phases of clinical trials. Drawing from these experiences, we present a set of guidelines that outline key principles and mechanics for game development in the clinical trial context. The mechanics and examples described can help coordinating center personnel and academic and clinical research organizations build games into existing clinical trial systems and successfully put gamification to work as an engagement tool, creating an enjoyable, engaging atmosphere, with the aim of improving coordinating center engagement and multisite team motivation.

## Methods

We conceptualized gamification as the translation of routine trial-related start-up tasks into an engaging interactive framework without introducing additional responsibilities for site teams. Existing trial activities such as protocol review, training completion, and document coordination were stylized as game tasks to enhance motivation and visibility of progress. The designs emphasized that gameplay would serve as a record and recognition of actual work completed at each site, with performance displayed through visual elements such as leaderboards, periodic newsletters, and webinar updates.

The game rules and objectives were aligned with the operational goals of the clinical trials to ensure fidelity to trial priorities. Five key considerations guided each design and build process as follows:

1. Theme (motifs and flow): Selection of a coherent narrative or motif to frame the game experience and maintain engagement
2. Scope (measures included): Identification of trial activities to be tracked and gamified, ensuring relevance to site performance metrics
3. Score and balance (play and win mechanics): Development of scoring systems that reward timely and accurate completion of tasks while maintaining fairness across sites
4. Recognition (rewards for performance): Implementation of tangible and intangible rewards—such as badges, certificates, and public acknowledgment—to reinforce achievement
5. Encouraging continued play: Strategies to maintain engagement over time, including periodic refreshing of game elements, milestone celebrations, and integration of social interaction features

This structured approach aimed to amplify intrinsic motivators—autonomy, mastery, purpose, progress, and social connection—while leveraging extrinsic incentives to reinforce engagement. By embedding gamification within existing workflows, the gaming interventions sought to improve engagement and motivation without increasing operational burden and improve multicenter trial productivity.

### Setting up the start-up game

Site start-up was a prime candidate for the creation of a single game that could be used across multiple trials. Game features, such as calculated scores and dashboards, were programmed into electronic data collection (EDC) systems or electronic Trial Master File (eTMF) systems by coordinating center personnel with minimal effort at the beginning of the site start-up phase. Additional coordinating center effort was required to program in reports and create game graphics to present standings, and later, to host award ceremonies at monthly webinars or through other means of communication, such as newsletters or online postings.

Once report templates were created, as coordinating center staff and site teams entered completion dates for start-up activities into the EDC or eTMF as part of their weekly start-up routines, relevant data was available to the coordinating center in real-time gamified formats. Site teams watched their points accumulate as they performed their start-up responsibilities. No additional effort was required on the part of the site teams. Monthly start-up webinars and award ceremonies highlighted top performers.

### Gamifying the active enrollment phase

The enrollment phase games were varied in format and similarly relied upon programmed reports from trial EDC and eTMF systems. We customized the gamification approach to address each trial’s specific needs for enrollment, screening, follow-up visits, and data entry and made further adjustments to overcome unique protocol challenges and periods of suboptimal performance.

The next section, “Gamification mechanics,” details how to use the five key considerations to design and construct a clinical trial game.

#### Gamification mechanics

##### Theme

A game theme is the subject matter, visuals, and motifs that define the central metaphor of a game. The concept of a game falls flat without a theme or player appreciation of the theme. It should be developed early because the theme influences how a game is designed, built, and played. The theme is the first impression the game will make on the players; therefore, the best theme is one that will engage players and transparently relate to trial goals. The theme could be related somehow to the trial intervention, name, or acronym or a current, well-recognized event, such as March Madness®, Winter Olympics®, World Cup®, or the America’s Cup®, all of which have been implemented with success. Whether a game is designed to cover the duration of a trial or applied to a particular phase, such as a site start-up, consider whether one theme will be suitable throughout the trial or whether different themes might better reflect trial goals or seasonal events as the trial moves along. It should be a storyline that helps site teams visualize work completed accurately and on time. The game becomes intertwined with the flow of trial tasks. Theme connects the life cycle of a game, from the minute it begins to the end, and requires clear presentation and feedback in service of the given objectives [11]. Effective use of visuals, engagement, and subject matter can enhance a team’s motivation to play a game. The theme and its flow should promote feelings of excitement and fun while providing a sense of satisfaction and progress; if it does not, then the game may not be effective [12].

##### Scope

Trial performance is multidimensional, and every trial will present its own unique performance indicators that can be highlighted with gamification. When designing a game, determine which trial activities or key performance indicators (KPIs) to highlight as measures of success and recognition for well-performing sites. Choosing metrics that cover the four major areas that influence the quality of a trial—recruitment, retention, protocol compliance, and data quality—is a good place to start. Gamifying enrollment is an obvious place to begin, but adding additional metrics could create a richer game. A comprehensive game will reward site teams for doing well across many desired trial behaviors, whereas a simple game will only pick a couple of metrics to emphasize. There are benefits and risks to both. A comprehensive game can provide detailed measures of overall site performance through the game but at the risk of diluting the game’s focus. A simple game, by contrast, provides an easy focus for sites to follow but risks de-emphasizing non-gamified tasks. Table 1 offers a selection of start-up and enrollment phase metrics to consider gamifying.

**Table 1:** Challenges and solutions for gamifying specific metrics

Metric	Challenge	Solution
**Site tasks**		
Time to delegation log completion	Sites with larger teams may take a longer time to collect all regulatory documents due to the volume of team members	When assembling a site team, sites should be discreet when making team assignments to avoid oversaturation
Time to training completion	Sites with larger teams may take a longer time to complete training due to the volume of team members	Sites should be discreet when making training assignments to ensure individuals are only assigned training for tasks they perform. Points can be awarded when all training is completed or per person. Per person should be weighted against the total site team size
Time to sIRB submission*	Metrics such as “Time to sIRB Approval” should be avoided as the time a submission takes in review is out of the hands of the site team	When utilizing start-up metrics for a game, choose measurable outcomes that can be tied directly to site team performance
Overall activation duration*	In site consortiums that begin start-up at varying time points, the first to activate may not necessarily represent the quickest to activate	Calculating the component metrics that contribute to the time spent in start-up allows for recognition and rewarding of discrete milestones as well as overall activation based on rate of completion, not just order
Number of screens	Screening data is susceptible to issues such as sites not entering screens or only entering screens for those who are randomized, so numbers may not accurately reflect patient populations	Ensure best practice screening log completions and accurate counts by adding point values to screens
Number of enrollments	If enrollment count is below the ideal threshold, or teams are de-prioritizing the study locally, diminishing enrollment numbers at the site level impacts the trial performance	Gamifying enrollments can incentivize teams to enroll more, getting the overall trial closer to enrollment goals
Screening and/or enrollment % of census	Varying site sizes will see higher or lower volumes of patients	If hospital census numbers are available from a study-wide cohort assessment, these metrics can be used to balance performance
Successful outcomes visits	Studies with complex and lengthy follow-up protocols may see higher rates of lost-to-follow-up and struggle with patient retention as time goes on	Emphasize the completion of follow-up visits during a lengthy follow-up period with points and/or bonuses for % completion overall
Participant adherence to protocol	Patient-reported outcomes and protocol adherence relies heavily on the site team’s communications with patients	This incentivizes site teams to follow up with enrolled patients to encourage compliance with trial outcome measures
Number of protocol deviations	Protocol deviations are inevitable. While some deviations are due to lack of site team attention, it is recognized that other deviations may be unavoidable for participant safety	Gamifying this metric motivates sites to follow the protocol as closely as possible. In trials with higher enrollment, the ratio of occurrence of unavoidable deviations compared to genuine issues of protocol performance will stabilize
Visits entered within 7 days	Documentation of visits within an appropriate time window can slip from a site team’s list if not done as soon as possible	Timely entry of follow-up visits reinforces Good Clinical Practice. Sites with fewer enrollments may have an easier time with this, and sites with lower socioeconomic status populations may have a more difficult time with follow-up adherence, which may need balancing
Queries answered within 7 days	Unresolved queries risk poor data quality and accuracy if not addressed as soon as possible	With robust monitoring, targeting query resolution as a metric is another best practice to maintain timely engagement with trial timelines. Sites with fewer enrollments may have an easier time keeping up with timely query resolution which may require balancing
**Institution tasks**		
Time to sIRB cede decision	When utilizing the sIRB model, there are various roadblocks to how and when site HRRP personnel cedes review to the sIRB	Gamifying this step can encourage site teams to actively monitor and assist the process as able
Time to contract redline version	Handled by contract office personnel, outside of the site team’s control/purview	Site teams should monitor progress and assist with communications to advance subaward review more quickly
Time to contract partial execution	Partial execution is handled by contract offices. Time from partial execution to full execution should not be gamified, as execution of subcontracts is in the control of the sponsor and may be withheld until sIRB approval is obtained	Site teams should monitor progress and assist with communications to partially execute (sign) the subaward quickly

^*^Indicates metric influenced by *both* site team and institution. Abbreviations: *sIRB*, single Institutional Review Board; *HRPP*, Human Research Protection Program. A selection of start-up and enrollment phase metrics to consider when gamifying trial tasks as well as selected challenges and solutions

**Fig. 1:**
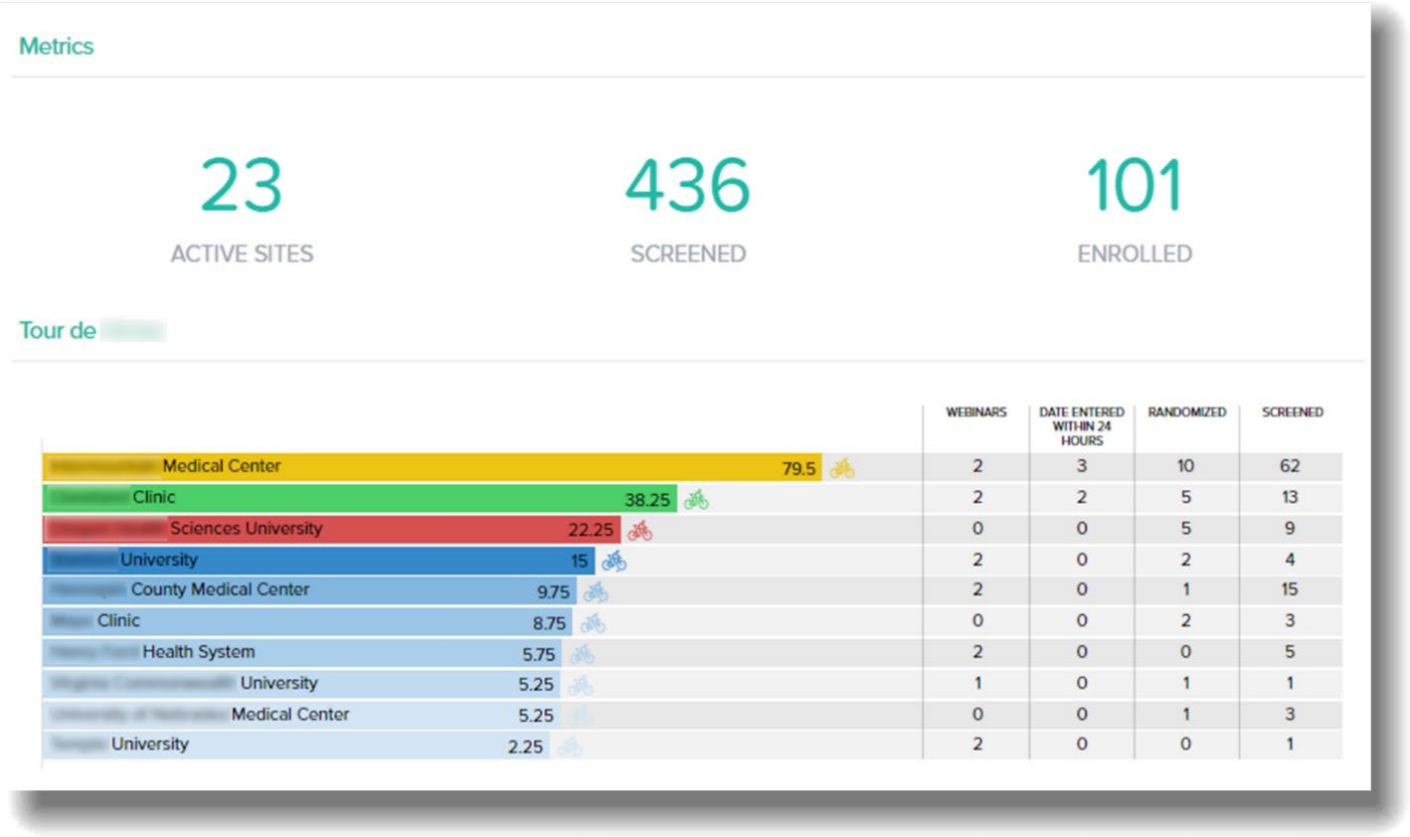
An attractive and easy to follow electronic data capture (EDC) scoreboard example for a trial using the Tour de France game. The scoreboard shows the relevant metrics and scores. In this trial, scoring was based on numbers screened, data quality, and webinar attendance

It is important to choose measures that can be converted into a data set with limited design time and building effort, especially if creating a game manually. The availability of EDC and eTMF systems can save time and expand computational capabilities for the metrics to be gamified. For example, time-based metrics might include the number of days early or late that a site was activated relative to a target date, and this calculation can be automated for ease of export (see supplementary materials for how to adapt various metric types into points for games.) Additionally, games built into an existing trial platform can provide feedback in real time as users interact within existing systems. Report generation features can be turned into easily accessible central scoreboards or dashboards with tabulated results and game features displayed (Fig. 1). Trial vendors and developers or staff developers (or other relevant technical assistance) can determine what can be implemented on existing data collection platform(s). In most cases, many metrics that will be of interest to gamify will already be regularly reported on by the coordinating center, as they are crucial to monitor the performance and success of the trial.

##### Score and balance

A scoring system that compares site performance metrics drives the internal mechanics of a game. Understanding the possible hurdles or difficulties in trial operations will help create an effective and relevant scoring system.

Metrics can be scored by conversion into points via weights or scored by achieving given thresholds.

**EXAMPLE**: A trial with a long follow-up period is prone to participants who drop out or are lost to follow-up. There are six follow-up visits over a 2-year period. While enrollments are a first key indicator, without the primary outcome measures, there can be no trial results. **SOLUTION**: Enrolling a participant is assigned 2 points. In addition, each successful follow-up visit is given a value of 1 point. Full completion of the follow-up period increases the number of points awarded to the site, totaling 8 points. Two of the four major performance areas are rewarded with retention and protocol performance via outcome collection outweighing the accomplishment of the enrollment.

This example features a point-based design. In such a system, multipliers, called weights, are applied to various metrics to balance them against each other to create a single harmonized total score from which sites are ranked. In a point-based weighted system, the primary challenge is how to harmonize the various performance metrics into a single score. There are several designs for assigning points, depending upon the type of data being scored: simple-weighted metric design, time-based design, and percent-based metric design. In a simple-weighted design, metrics are scored based upon the number of events scored and weighted for relative value (e.g., screens, enrollments). In time-weighted designs, points are calculated based on the time between a start and finish (e.g., time from activation to first randomization). Percent-based designs bundle and normalize the total number of events of that type that do occur against the number that could occur (e.g., % of queries answered within 7 days). Instructions, equations, and examples of point systems are detailed in the supplementary materials.

Alternatively, thresholds may be used to create the structure of a game. In a threshold points system, the above example could be adapted so that a site team would earn a point by meeting a monthly enrollment goal and an additional point by completing all outcome visits scheduled for that month. Sites that complete all monthly goals naturally pull ahead of sites that do not. Threshold games tend to be simpler to score but offer less differentiation between sites and are therefore more susceptible to ties.

Scoring elements can be utilized to incentivize desired behaviors outside of the four major areas that influence the quality of a trial. Bonus points can be incorporated to promote leadership and engagement with the coordinating center and other site teams. Such things include but are not limited to credit for writing trial newsletter articles, attending trial-related webinars and/or giving presentations at webinars, submitting posters for annual meetings, presenting at local grand rounds, etc. Be creative when rewarding site investigators and teams for their hard work and going beyond traditional site team responsibilities, such as serving on the steering or executive committees or attending annual meetings. Find entertaining lesser events or accomplishments to recognize at ceremonies and meetings. Find ways to bring more winners into the circle. Plentiful and diverse acknowledgements make gamification more inclusive and social.

Game balance is the design concept that governs the equilibrium of strategies and the game’s reward elements. Good balance prevents a single strategy from unfairly overshadowing other strategies and ensures that the players have a fair and meaningful experience. How one chooses to target and weigh performance metrics determines the balance of the game. Performance indicators that are more difficult to execute can be valued (weighted) more than easier activities. There are many approaches to game balance, and the methods chosen should reflect the specific goals for the game. In a traditional game, the goal of game balance is to ensure that the best player wins every time. However, remember that the goal of gaming in a clinical trial is to enhance site engagement and performance across *all* study sites. One might initially think about rewarding only the best-performing site(s), but lack of recognition for other sites might cause engagement to suffer. It is helpful to think through situations that may arise that impact game balance; for instance, what happens if one site has an insurmountable lead? What if sites have clear differences in enrollment capability? How can underdog sites be rewarded without making the frontrunner sites feel unfairly penalized for their superior performance?

In most trials, there will be a naturally wide range of site performance. Sites intrinsically have structural characteristics that will likely affect how well they are able to achieve one or all metrics that a game includes. This could be as simple as differences in the size of the patient population, patients’ socioeconomic status (especially important for outcomes), to differences in coordinator and departmental support. Performance also may naturally be impacted by temporal circumstances such as a lack of team coverage, personnel changes, and administrative burdens. In a simple game, such as one that only tracks enrollments, some sites may not be able to keep up, no matter how much effort they dedicate to enrollment. Therefore, an important method to account for such differences is to consider both scaling and nonscaling metrics when balancing a game. A scaling metric is one that always increases with more events, such as an enrollment count. A nonscaling metric has a maximum value no matter how many instances occur, such as percentage-based metrics (% of queries answered within 2 days, etc.). Scaling metrics can favor sites with certain characteristics over others, while nonscaling metrics are achievable equally across all sites and may be easier to achieve at smaller sites. Table 1 contains a list of example metrics to include and important considerations when using those metrics for proper balance. Additionally, it may be helpful to choose a theme that groups together multiple sites as a team (e.g., boat races where multiple sites make up a single rowing team). The teams may then be balanced by the metric(s) most likely to cause an early imbalance (i.e., combine high enrolling with lower enrolling sites), allowing each team an even playing field. Such team dynamics can also foster collaborative dynamics and sharing best practices.

##### Recognition

Games naturally afford fun ways of displaying progress and providing recognition for achievements. Reward systems are essential to include in the theme’s design. Attributes describing progress should relate to the game theme and provide feedback relative to the tasks, providing opportunities for team members to fulfill their intrinsic need to feel competent and accomplished [8]. Such attributes might include progress bars, levels reached, or a collection of accomplishment badges.

Games that foster interest in an individual and provide enjoyment are intrinsically motivating, but external rewards such as acknowledgment can be equally effective. Such rewards are symbolic of already existing tasks and goals and are designed to elicit a sense of competence and autonomy, especially in relationship and comparison to others [3]. In addition to providing all site teams with access to the same leaderboard to recognize top performing sites, site teams can also be awarded long-lasting and displayable certificates, plaques, and trophies. Sites can be encouraged with team-building rewards, such as educational luncheons hosted at the site or educational grants. It is important to choose reward structures acceptable to local Institutional Review Boards (IRBs). Institutional guidelines will govern what is acceptable for site researchers and staff to receive. Some tangible awards, especially beyond simple acknowledgment, may be considered undue bonuses or incentives. Typically, acceptable awards will be capped to a certain minor dollar amount, making adding such awards to your game inexpensive. Even so, awarding gift cards, educational luncheons, or educational grants as an award for exceptional performance should be decided early and built into budgets.

##### Encouraging continued play

When site teams begin to play a game, the novelty of it is usually interesting enough to draw their attention. However, for long-term games, it is important to continuously promote gameplay. Some studies have shown that sustained gameplay is short-lived except for those who are competitively inclined, and that those who are not in the top performer circle will engage with the system less often [13]. Several strategies can combat this. Games can be conducted in multiple segments or as shorter discrete separate games. In individualized games, a close-out award ceremony for early winners can end one race and begin anew, giving sites a second chance to win in the new game. If using a team-based model, recombining winning teams with lower scoring teams can rebalance the game while starting a new race. This is especially encouraging to site teams that have experienced setbacks unrelated to the trial itself, such as a valued team member going on family leave.

Progress is one of, if not the most, important variables that impacts motivation and engagement [8]. The difficulty level of a game also significantly impacts whether a player continues playing long term, although a game’s ease of use does not necessarily indicate scoring points will be easy [14]. Consider what aspects of the trial will be most difficult for the sites to achieve and reward them accordingly. The game’s theme can highlight the most difficult tasks with the most impressive metaphors, from finishing a difficult turn in a race quickly to hitting a curveball for a homerun.

Elements such as personalization of team accomplishments and access to standings and evaluative feedback will encourage engagement and can make or break sustained interest in a game. One study found that evaluative feedback had the strongest effect on increasing a player’s likelihood of playing the game again. Phrases like “You completed the task rather quickly” were correlated with players wanting to continue playing the game long term, while negative feedback prompted players to play the game again in the immediate future, but not long term [9]. Even if the game is not high-tech enough to be immediately interactive, coordinating center staff should deliver feedback regularly. Use available communication tools, from trial-wide notices on websites, leaderboards, webinars, and newsletters showing the current standings, to special emails and thank-you letters personalized to individual teams or persons. In addition to lauding the game winners, give special recognition for outstanding performance in individual areas. Additionally, recognize individual outstanding site team members for their contributions to their site’s performance. There is no limit to appreciation and recognition.

## Results

We successfully implemented gamification strategies for nine Phase II and III trials across various therapeutic areas for both the start-up and enrollment cycles (Table 2). The same start-up game was played for seven trials; given the similarity of start-up tasks across these trials, we were able to create a single game that effectively served multiple studies. Ten customized games were used by 9 different trials during the enrollment cycle; the cardiology trial used 2 games during its enrollment phase. In all, 17 clinical trial games were run with over 300 distinct site teams having played at least one game with multiple scoring methods and features.

**Table 2:** Trial characteristics and game features by types of games played

Name of game	No. of trials playing game	Trial phase	Therapeutic areas	Trial cycle	No. of sites playing game	Scoring method	Included features
**Mount Everest**	7	Phases 2/3	Neurocritical care	Start-up	47	Points	Webinars (live sessions and recordings) Award ceremonies Trophies Certificates of achievement Conference support
			Neuroimmunology		43		
			Critical care medicine		43		
			Neurosurgery		8		
			Cardiology		86		
			Infectious disease		9		
			Neurosurgery		21		
**World Cup®**	2	Phases 2/3	Neurocritical care	Enrollment	82	Points	Webinars (live sessions and recordings) Newsletter Luncheon rounds
					91		
**Rowing competition**	1		Neuroimmunology		43	Points	Webinars (live sessions and recordings) Award ceremonies
**Tour de France®**	1		Critical care medicine	Enrollment	40	Points	Live dashboard Award ceremony
**Build-Your-Characters with Unique themes**	4	Phases 2/3	Neurosurgery	Enrollment	21	Threshold	Webinars (live sessions and recordings) Award ceremonies Local vendor gift cards
			Pediatrics		14		
			Neurosurgery		12		
			Infectious disease		11		
**March Madness®**	1	Phase 3	Cardiology	Enrollment	71	Single metric	Weekly newsletter
**Olympics®**	1	Phase 3	Cardiology	Enrollment	81	Points	Newsletter website leaderboard Award ceremonies

Investigators and sponsors incorporated gamification in Phase 2 and 3 trials, across various therapeutic areas, during start-up and enrollment cycles. A single Mount Everest game was played during the start-up cycle for seven trials. Ten games were used by 9 different trials during the enrollment cycle; the cardiology trial used 2 games during its enrollment phase. In all, 17 clinical trial games were played, with over 300 distinct site teams having played at least 1 game with multiple scoring methods and features

### Three trial games detailed to share

The ensuing games can be used partially or in their entirety to aid in the construction of clinical trial games. The Mount Everest game was designed for the trial start-up phase. The Tour de France game was developed and utilized in a trial that exceeded its projected enrollment timeline but was halted early for lack of efficacy. The Superhero game was played by teams in a trial that successfully met its enrollment goals and completed on time.

#### Mount Everest game

**Fig. 2:**
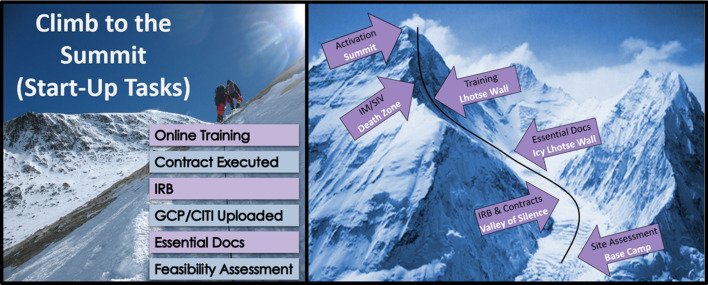
An example of transforming a start-up metric into a game and reward system is to use the “Mount Everest Climb,” an approach designed to (1) recognize and reward site adherence to a timed start-up plan and (2) assist sponsors and coordinating center managers and monitors track the start-up process in a fun and competitive way. “The Summit” (activation) is reached after the team makes it through an exhilarating climb past the “Valley of Silence” (IRB & contracts), scaling the icy Lhotse Wall (regulatory [essential] documents), the final Lhotse Wall (training), and finally the “Death Zone” (investigator meeting or site visit). At the end of the climb, an award ceremony can be held at an investigator meeting, site initiation, or through a remote broadcast, to recognize the site teams that first completed required site-activation tasks and otherwise have successful accomplishments to share. Recognition goes a long way

The Mount Everest game was built into a dedicated start-up management platform which allowed for easy calculation at the coordinating center of the time-based metrics in all seven trials. Just like scaling the mountain, we built the game with discrete stages and parallel challenges for sites to quickly reach the mountain summit and activate (Fig. 2). The climb was designed with multiple base camps as climbers trekked onward, and activities were divided into three monthly base camp milestones. Site teams were timed and scored based on when they began start-up (left base camp). The theme tracked start-up activities easily, regardless of whether sites left base camp at separate times, in multiple groups, or all at once. Several balance and scoring considerations were incorporated into the game. Point totals expressed an overall rate of multiple task completions rather than the single measure of which site team crossed the finish line first. While activation was the end goal, multiple common barriers exist in every activation timeline. Therefore, the game was designed to reward individual start-up tasks along the way (Table 3). The ultimate step of activation was assigned a small point value so as not to disadvantage otherwise well-performing site teams that might be delayed by tasks not in their direct control at the institution level. Tasks that were risk factors directly within the site team’s control were assigned larger point values. Completion of site team tasks, such as drafting initial IRB-related documents, submitting and routing approvals, collecting personnel-level regulatory documents, and completing training requirements, was emphasized. Smaller point values were assigned for completion of institutional IRB or Office of Research Administration (ORA) deliverables beyond the site team’s control (e.g., obtaining partial execution of a contract from a busy contract management office). Such tasks were still included, however, to incentivize site teams to follow up with their institutional offices to ensure such tasks do not get lost. To prevent any single task from having an outsized effect on a site’s overall point total, bonus points given per day early or points deducted per day late were capped at 14 days. Tasks assigned to a greater initial point value were assigned to a correspondingly higher weight on the bonus/penalty points per day. Typical Mount Everest time-based metric distribution and weights are detailed in Table 3 and further in the supplementary materials.

**Table 3:** Applying game points to tasks for a start-up game

Metric	Goal duration (days)	Base points for completion	Bonus points per day (late/early)
Protocol available to local context Questionnaire (LCQ) first draft	21	5	−0.4/+0.4
Protocol available to site-specific consent Information (SSCI) first draft	21	5	−0.4/+0.4
Contract available to partially executed	42	10	−0.7/+0.7
Regulatory document templates sent to delegation log circulation	35	5	−0.4/+0.4
Delegation log circulation to finalization	14	5	−0.4/+0.4
Regulatory document templates sent to site-level regulatory documentation completion	56	15	−0.9/+0.9
Regulatory document templates sent to personnel regulatory documentation completion	56	10	−0.7/+0.7
Training available to training completion	77	10	−0.7/+0.7
Total site activation duration	90	1	−0.2/+0.2
*Weekly meeting attendance		1 per meeting attended	N/A
*Monthly webinar attendance		1 per webinar	N/A

^*^An Accelerated start-up program can be used in conjunction with this game design, as it features weekly check-in meetings with the coordinating center and monthly educational webinars covering important start-up topics. A single point can be rewarded for each meeting and webinar attended. Site start-up is a prime candidate for the creation of a game that can be used across multiple trials. Various points are earned by meeting task deadlines, and bonus scores can be awarded or subtracted for early completion or missed deadlines, respectively

#### Tour de < Your Trial Name Here > (AKA, France) game

**Fig. 3:**
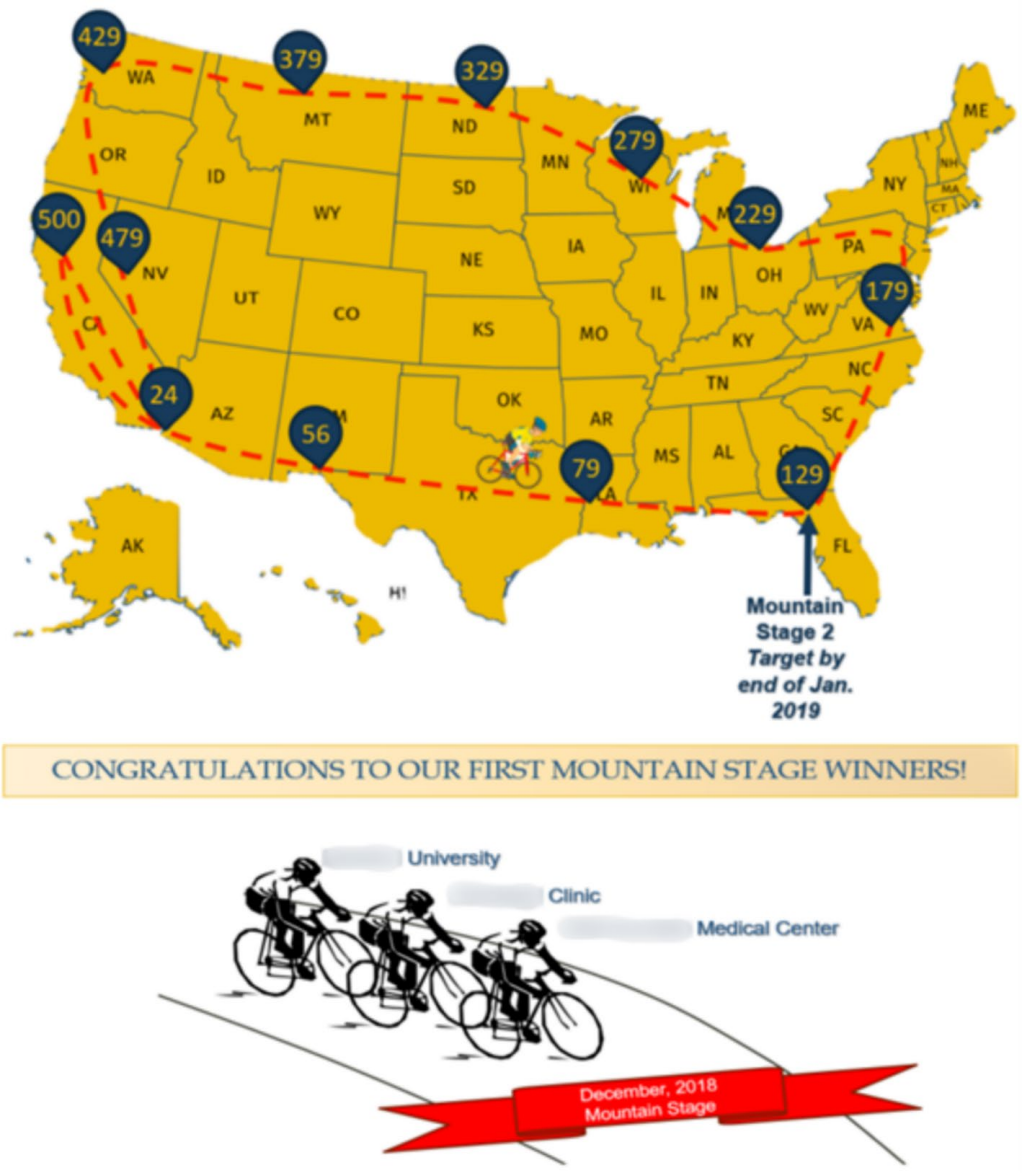
The Tour de France style game is a point-based design for gaming during the trial enrollment cycle and can be easily programmed reports from trial electronic data capture (EDC) systems. Episodic subgames can be programmed for any given metric for monthly bonuses that can allow trailing teams to catch the leading teams

This was a selective game, focusing on a few key metrics. Professional cycling and the Tour de France® concepts were adapted to show monthly enrollment progress by cycling across a map in a race to recruit participants towards the trial’s enrollment goal (Fig. 3). This game theme utilized both scaling and non-scaling metrics (see also supplementary materials) while remaining selective and simple to understand to engage the sites. Creating a balance between scaling and nonscaling metrics also ensured that site teams with larger patient volumes did not have an advantage in the game.

To encourage continued play, in addition to the overall cross-country cycling event, there were episodic “Mountain Stage sprints” programmed in during certain trial months, when additional bonus points were earned by being the leader in any given category for a month (Fig. 3). These bonuses targeted historically difficult months for enrollment and meeting special milestones. For example, bonus points for enrollment were awarded during holiday-laden months like December or for enrolling the 100th patient. These special sprints encouraged activity and allowed trailing site teams the opportunity to catch up to the leading teams. Additionally, sprints were tailored to target specific challenges, such as lagging data entry or follow-up visits missed.

#### Superhero game

**Fig. 4:**
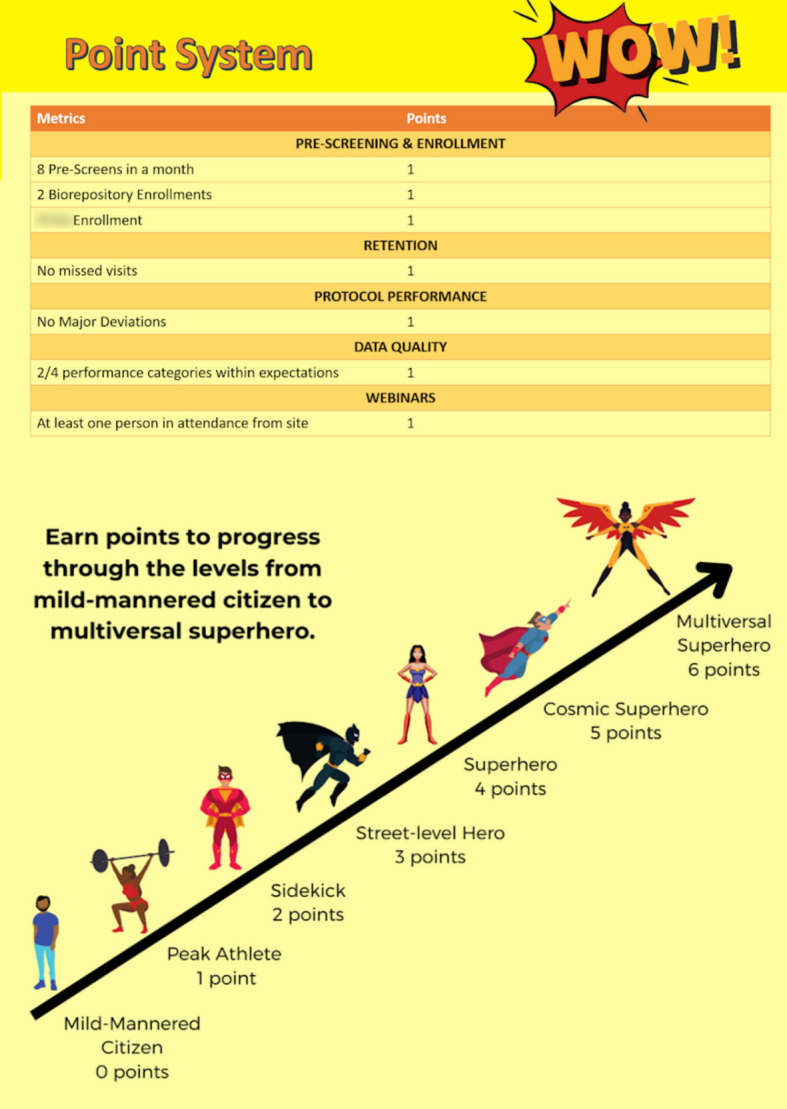
The Superhero game is a threshold-based design and is useful when automation within an electronic data capture (EDC) is not feasible or there is not a central platform to display results. Scores can be extracted from data coordinating center reports and displayed monthly through website postings, monthly webinars, or individual communications with site teams, affirming that gamification can occur manually with minimal technical needs

This game offered a threshold-based design when direct programming into an EDC was not feasible (Fig. 4). The coordinating center staff utilized pre-programmed data center reports to manually track defined site performance thresholds. Each threshold increased the site superhero “level” for a given month. Sites that did not meet performance goals were described as “mild-mannered citizens.” Sites meeting all goals were “Cosmic Superheroes,” and various other levels of superhero were in the middle. In addition, badges were awarded to sites for exceptional performance beyond the given thresholds. Sites were scored month to month to give exceptionally well-performing sites in any given month proper recognition for performance, leveling recognition with sites that consistently performed better (as might be the case in a points-based system where sites are scored cumulatively). Presentation of the game leveraged existing and planned trial-wide communications. This game format was designed to offset limited data access, affirming that gamification can occur manually with minimal technical needs.

## Discussion

Gamification is a tool for central trial staff to better engage with site coordinators, principal investigators, and other site staff responsible for patient recruitment, retention, protocol compliance, and data quality during a clinical trial. While there is no guarantee that gamification will result in a faster or more productive trial, there are benefits to using games. When sites feel disengaged or disheartened, games can be used to encourage collaboration and a renewed sense of commitment to trial goals. Well-designed games can turn a trial’s burdens into exciting challenges or reduce overwhelming goals to smaller incremental goals that can be achieved more easily. In this way, gamification can change how trial burdens are perceived and feelings of disconnection can be relieved. Gaming also can create opportunities for team recognition, friendly competition, and fun. Turning routine tasks into a structured game format not only adds an element of fun but also encourages engagement and competition between site teams who may know one another. In one trial, sites said they did not care where their site ranked if they were not falling behind friends on other site teams.

Through gamification as a social interaction, trial leaders can acknowledge site teams for their autonomy and mastery in meeting deadlines and goals and for making a difference with their individual site progress. Gamification is a way to show how successful each team is, which can make a difference to both local colleagues and other site teams. There is a natural desire to see results in the form of small wins (weekly if not in real time) that can ignite joy, engagement, and creativity. And of course, the need to belong and to be praised is always present. This can be particularly helpful in prolonged trials where both perseverance and persistence are required. Project managers and coordinating center leaders can fulfill the role of cheerleaders, giving kudos to site investigators and coordinators for beating benchmarks and using creativity to get things done.

Gamification can be included in any phase of a clinical trial, as within each period there are activities that occur in quantified amounts and in identifiable, defined durations. Gamification can be used as a tool to better connect and engage coordinating center personnel and site teams in general or to focus on specific trial goals or tasks that are subject to completion risk. For games conducted during the enrollment period, designing the EDC to quickly tabulate KPIs is paramount, not just for gamification’s sake but for identifying trial delays or deviations as they happen.

Done well, a game will have a balanced and weighted game scoring system for aspects of the trial that are both the easiest and the most difficult for sites to achieve. Whether they involve securing institutional approvals, enrolling rare populations, or accurately performing a complex protocol, sites should easily see their accomplishments, rewards, and displays of recognition. Finally, games should not add burdens to site teams. Games should be built using metrics already collected on trial data platforms where games can be tabulated and scored automatically at the coordinating center or on the data platform. This way, site teams are spectators of their own accomplishments achieved by doing the day-to-day routines of conducting a clinical trial. As a final suggestion, study leaders should regularly review the performance of the game itself (not just the players) for internal consistency and ease of use and potentially survey the satisfaction of the participating sites for ways to improve the clinical trial game elements and process.

### Limitations

Throughout motivational literature, responses to workplace gamification vary depending on attitudes towards gamification, the purpose of the game, the type of game, and individual drivers of motivation [3]. Competition can demotivate noncompetitive personalities [15, 16]. However, team-based competitions, such as these clinical trial workplace games, are more likely to promote work group connectivity and social connection [17]. Also, designing a game to be played by a site team or regional team is a social element that favors motivational benefit. Gamification webinars, newsletters, and award ceremonies highlight cooperation and link players into a common cause, adding appeal to players with varying achievement orientations and may even mitigate potentially negative effects on noncompetitive personalities [18]. Clinical trial gamification also differs from non-trial workplace gamification in multiple nonthreatening ways. The game is voluntary, not driven by employers or dependent on budgets or salaries, and these types of games award recognition for completion of tasks but not the quality of the task completion (as in a quality assurance or performance review). Therefore, negativity resulting from imposing gamification on employees is absent, while the nonthreatening benefit of a game providing a clear pathway for milestones and getting to the “next level” is present.

Certain metrics can be susceptible to manipulation. For instance, a screening-to-enrollment conversion ratio may seem advantageous to reward, thinking that sites randomizing a higher proportion of screened patients are doing a better job. However, some sites may consider omitting screenings from their logs to enhance their ratio artificially. Although it may be uncommon for site teams to engage in such manipulation, it remains essential to monitor these behaviors closely and to avoid incentivizing metrics that could lead to unintended consequences.

If game elements cannot be built directly into an EDC or eTMF, then gamification without undue burden becomes limited to KPIs that will be most easily accessible in weekly or monthly reports. Investigate which metric reports are available to support game creation with minimal burden on coordinating center staff.

Tapping into an existing communications framework is ideal. Consider how frequently game scores or results can be updated and how results can be disseminated to the sites. Will there be regular site communications where scores/results/accolades will be highlighted? If an EDC dashboard is unavailable, game results can be posted to a study website and updated as often as possible. The easiest path is to use whatever standard site communication methods are used as part of a trial’s communication plan to display the game results. If an existing communications network is not already present, additional effort will be required to build such a structure and plan.

And last, although our previous research suggests that site teams who enjoyed participating in our start-up games performed faster, and all games presented here were associated with successful trials, further research is necessary to better understand the performance impact of gamification in a more robust manner.

## Conclusion

Incorporating games into clinical trials signals to site teams that the coordinating center staff is committed to fostering engagement, and this enthusiasm can thereby enhance overall trial engagement. The mechanics and examples provided have been successfully implemented across a variety of trials, and we hope that sharing these gamification strategies will inspire their use as tools for boosting trial engagement in future studies within the translational science workforce. Additionally, we aim to promote a spirit of fun and creativity among clinical trial teams. While existing data suggests that site teams who enjoy participating in these games perform tasks faster during the start-up phase, further research is in progress to quantify the impact of gamification on site team performance following activation.

## Supplementary Information

Supplementary material 1. Detailed methods and considerations for game scoring and balance. Method 1: Simple Weighted Metric. Table 1: Simple Weighted Metric Example. Method 2: Time-Based Metric. Table 2: Time-Based Metric Example. Method 3: Percent-Based Metric. Table 3: Percent-Based Metric Example. Table 4: Scoring rules for the Tour De France Example as seen in Figure 1. A simple points game with scaling and non-scaling metrics. Table 5. List of start-up and enrollment period metrics and suggested methods to calculate them. The weight portion of the metric is a simple arbitrary multiplier used to balance the impact of the metrics in a game. Table 6: Scoring method of a threshold formatted version of the example Tour De France game. In this version of the game, the game is scored and presented monthly rather than scored live via a calculated dashboard.

## Data Availability

All data generated or analyzed during this study are included in this published article (and its supplementary information files).

## Abbreviations

EDC: Electronic data capture
eTMF: Electronic Trial Master File
HRPP: Human Research Protection Program
IRB: Institutional Review Board
KPI: Key performance indicator
ORA: Office of Research Administration
NCATS: National Center for Advancing Translational Sciences
NIA: National Institute on Aging
TIC: Trial Innovation Center
