# Supplementary material for: Building games into multicenter clinical trial systems to boost trial engagement
Source: Trials. 2026 Feb 13;27:218. doi: 10.1186/s13063-026-09498-6 (PMC13005411; doi:10.1186/s13063-026-09498-6)
Supplement: Supplementary file 1 — Supplementary material 1. Detailed methods and considerations for game scoring and balance. Method 1: Simple Weighted Metric. Table 1: Simple Weighted Metric Example. Method 2: Time-Based Metric. Table 2: Time-Based Metric Example. Method 3: Percent-Based Metric. Table 3: Percent-Based Metric Example. Table 4: Scoring rules for the Tour De France Example as seen in Figure 1. A simple points game with scaling and non-scaling metrics. Table 5. List of start-up and enrollment period metrics and suggested methods to calculate them. The weight portion of the metric is a simple arbitrary multiplier used to balance the impact of the metrics in a game. Table 6: Scoring method of a threshold formatted version of the example Tour De France game. In this version of the game, the game is scored and presented monthly rather than scored live via a calculated dashboard. [file 13063_2026_9498_MOESM1_ESM.docx]

**Supplementary Materials: Detailed methods and considerations for game scoring and balance**

**Points-Based Games vs. Threshold-Based Games:** In a points-based system, the primary challenge in constructing the scoring mechanics of your game is how to harmonize various metrics into a single score. Multipliers, called weights, are applied to various metrics to balance them against each other, ideally according to their relative value to the success of the trial. Points based games are ideal when you are looking to create a nuanced game that rewards many different site goals and behaviors in a single score. They work best when data is readily and easily exportable and calculation can be automated, as having a diverse set of gamified metrics with different calculations can create unnecessary burdens otherwise. In the event the game and necessary calculations are built into the EDC, the game can even feature a live scoreboard, as points are tied to the events as they happen. Alternatively, threshold-based games are ideal when calculation of this sort is infeasible. They are most readily created out of existing KPI goals and are scored by simply tallying if a site met each given goal per each scoring period. Games of this sort can be simpler to create and manage but are more prone to ties. They are best scored at given discrete intervals (i.e., monthly) and cannot feature live dashboards.

**Points-Based Game Metric Scoring Methods and Equations:** There are several methods for scoring the components of a game in a points-based design, owing to the intrinsic properties of the metric in question. Each type of one has its own benefit for the balance of your game.

**Method 1: Simple Weighted Metric.** For most metrics, a discrete number of event occurrences can be counted with a given weight to scale the metric to be worth an appropriate number of points within the game. Weight is defined as any arbitrary multiplier applied to data to achieve a score or score component. This method is straightforward and works best for metrics such as screened or enrolled patients and completed outcomes visits. In the simple-weighted metric Supplementary Materials Table 1 example, a trial required intensive screening around narrow inclusion and exclusion criteria. The game builders assigned screening efforts a weight of .25, and enrollment successes a weight of 4. Every screened patient counts towards the total point count, but the team with the more efficient conversion rate (from successful screened to a successful enrollment), earns more points. This type of scoring rewards effectiveness as well as sheer numbers.

**Supplementary Materials Table 1:** Simple Weighted Metric Example

| **SITE** | **ENROLLED** | **SCREENED** | **POINTS/ENROLLMENT** | **POINTS/SCREEN** | **TOTAL** |
| --- | --- | --- | --- | --- | --- |
| **A** | 1 | 20 | 4 | 0.25 | 9 |
| **B** | 3 | 4 | 4 | 0.25 | 13 |

***Score = Metric x Weight:*** Site A and B screen and enroll a different number of patients. Shown is an example of how each would score with the given weighting. Site A screens 20 patients and enrolls 1, giving the site 9 points for screening and enrollment (1*4 + 20*.25 = 9). Site B screens only 4 patients, but enrolls 3, scoring 13 points (3*4 + 4*.25 = 13).


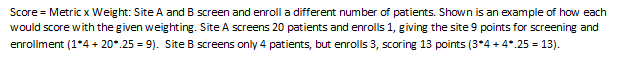


**Method 2: Time-Based Metric.** For metrics involving specific times with deadlines, i.e., when an activity or study event must be completed, it may occur before or after the specified deadline. In these situations, a bonus or point deduction can be calculated into the completion timeliness scoring, even to the degree of how early or late the activity was completed. This method of scoring is best applied to sparse or unique events that can be assigned to a reasonable but ambitious goal, such as the time taken for regulatory documents to be on file or contract execution (see time-based example below). In these cases, a base score can be applied to the expected completion date; then, points earned are modified at completion by the number of days before (bonus points) or after (deducted points) the expected date (Supplementary Materials Table 2). This type of scoring rewards getting the task done and additionally recognizes those who accomplish the task more effectively. The site contract negotiation process is a good target for this type of scoring: the goal is set for contract negotiation and execution and measured as the time between a contract being received by a site to the time of partial execution at the site. Do not use this type of scoring to incentivize participant-related activities, as completing a clinic visit too early could be detrimental to participant safety or data integrity. For this metric type, it is typically necessary to implement an upper and lower bound to either the number of days the weight will apply or the final points a site could earn on any given task. In the Supplementary Materials Table 2 example, suppose at site A, the contract negotiation happens slower than the 49-day goal and takes 103 days. While this is common, it is certainly not ideal. By the formula in Supplementary Materials Table 2, the site would score 10-(54*0.5) for a total of -17 points. A lower bound, in this case, prevents a single poor metric from invalidating other potential successes at a site. Conversely, if a site completes a given task much faster than expected, an upper bound prevents a single metric from catapulting a site team on to win when many of its other metrics might not be as stellar. Whether choosing to limit the number of days a weight can apply or cap the possible scores for any given metric, consistency in application is recommended to avoid creating too convoluted a system.

**Supplemental Materials Table 2:** Time-Based Metric Example

| **SITE** | **GOAL CONTRACT NEGOTIATION DURATION** | **ACTUAL DURATION** | **POINTS FOR TASK COMPLETE** | **POINTS PER DAY EARLY/LATE** | **TOTAL** |
| --- | --- | --- | --- | --- | --- |
| **A** | 49 | 51 | 10 | 0.5 | 9 |
| **B** | 49 | 41 | 10 | 0.5 | 14 |
|  |  |  |  |  |  |

***Score = Metric + (Days Early or Late) x Weight:*** Site A and B complete their contract negotiations processes at different speeds. For this example, an expectation of 49 days is selected as the deadline to complete a partially executed contract. The expected date is given a weight of 10 points, with a bonus or penalty of +0.5 points per day early or –0.5 per day late. Site A completes the partially executed in contract in 51 days, earning 9 points for this task during startup (10 - (2*0.5)). Site B completes the task 8 days early, earning 14 points (10 + (8*0.5)).

**Method 3: Percent-Based Metric.** Continuous events that occur in significant numbers and must be well-timed should be expressed as a weight applied to a percentage of those events that occur within the goal period. The most notable example of this type of metric is data entry timeliness (Supplementary Materials Table 3). In contrast to large, rare events, data elements are numerous, and total counts can vary per participant. Scoring on a per-data-element basis would be impractical and incredibly difficult to perfectly balance. To simplify this, a weight can be applied to the percentage of needed data elements entered within a certain number of days of a given event. Such metrics are normalized to the total number of events of that type that occur. This makes them especially useful to score the quality of a process that might occur at different frequencies across sites or even across participants. Rounding is typically necessary when assigning points to this type of metric, as ratios of event frequency rarely result in manageable numbers for a scoring system. If there are no events to score, then use good judgement as to that event’s importance. For query compliance metrics for example, having no queries typically means perfect data entry, and should be awarded accordingly. If there are no outcome visits within a given time, as in the example in Supplement Table 3, the site should score zero.

**Supplemental Materials Table 3**: Percent-Based Metric Example

| **SITE** | **TOTAL NUMBER OF VISITS** | **VISITS ENTERED**  **WITHIN 48 HOURS** | **PERCENT COMPLETION** | **POINTS FOR 100%** | **TOTAL**  **(ROUNDED)** |
| --- | --- | --- | --- | --- | --- |
| **A** | 45 | 41 | 91.11 | 10 | 9 |
| **B** | 102 | 73 | 70.19 | 10 | 7 |

***Score = (number of events occurring within desired timeframe/total events) x Weight:*** Sites A and B have differing rates of data entry: site A has fewer outcome visits but is better able to stay on top of data entry. For this example, the expected goal is to complete data entry within 48 hours of a visit and is worth up to 10 points in the trial’s game. Site A completes 41 of 45 data fields on a subject’s case report form within 48 hours and scores 9 points for this metric (41/45 * 10 = 9.111). Site B, by contrast, has more patients, and therefore more data entries. However, this added entry has caused a decreased rate at which they are able to enter data within the 48-hour goal, completing 73/102 visits within 48 hours and earning 7 points (73/104 *10).


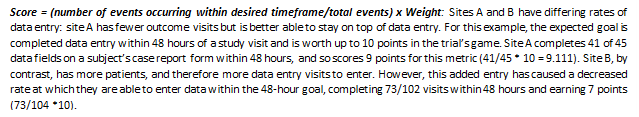


**Supplementary Materials Table 4**: Scoring rules for the Tour De France Example as seen in Figure 1. A simple points game with scaling and non-scaling metrics.

| **Tour De France Metric** | **Tour De France Weight** |
| --- | --- |
| Screening | .25 points per screen |
| Randomization | 4 points per randomization |
| Data Entry: % Of Data Entered within 24 hours of a visit | Normalized to 10 points for 100% |
| Webinars | 1 point per monthly webinar attended  5 points for presenting |

**Supplementary Materials Table 5.** List of start-up and enrollment period metrics and suggested methods to calculate them. The weight portion of the metric is a simple arbitrary multiplier used to balance the impact of the metrics in a game.

|  |  | **Metric** | **Metric Type** | **Sample Calculation** |
| --- | --- | --- | --- | --- |
|  | ***Start-Up*** | | | |
|  | IRB | Time to IRB Submission | Time-Based Target | Completion Weight + (Goal Duration - (Date of IRB Submission – Date of Protocol Receipt) * Weight) |
|  |  | Time to single IRB Cede Decision | Time-Based Target | Completion Weight + (Goal Duration - (Date of Cede Decision – Date of Cede First Contact) * Weight) |
|  | *Contract* | Time to Contract Redlines | Time-Based Target | Completion Weight + (Goal Duration - (Date of Redlines returned to central ORA – Date of Subaward Release) * Weight) |
|  |  | Time to Contract Partial Execution | Time-Based Target | Completion Weight + (Goal Duration - (Date of Partial Execution – Date of Subaward Release) * Weight) |
|  | *Regulatory and Training* | Time to Delegation Log Completion | Time-Based Target | Completion Weight + (Goal Duration - (Date of Log Completion – Date of DOR template Receipt) * Weight) |
|  |  | Time to All Training Completion | Time-Based Target | Completion Weight + (Goal Duration - (Date of Training Completion – Date of Training Available) * Weight) |
|  | *Overall* | Activation Duration | Time-Based Target | Completion Weight + (Goal Duration - (Date of Activation – Date of Protocol Available) * Weight) |
|  | ***Enrollment*** | | | |
|  | *Recruitment* | Number of Screens | Simple | Number of Screens *Weight |
|  |  | Number of Enrollments | Simple | Number of Enrollments *Weight |
|  |  | Screen % of Census | Percent-Based | (Number of screens / Number of patients seen with the condition of interest) *Weight |
|  |  | Enrollment % of Census | Percent-Based | (Number of Enrollments / Number of patients seen with the condition of interest) *Weight |
|  | *Retention / Protocol* | Successful Outcomes Visits (Scaling) | Simple | Number of Outcomes Visits within Window * Weight |
|  |  | Successful Outcomes Visits (Non-Scaling) | Percent-Based | (Number of Outcomes Visits within Window / Total Number of Visits) *Weight |
|  |  | Participant Adherence to Protocol (Scaling) | Simple | Number of Subject dependent entry events * Weight |
|  |  | Participant Adherence to Protocol (Non-Scaling) | Percent-Based | (Number of Participant Dependent Entry Events / Total Number of Possible Events) * Weight |
|  |  | Number of Protocol Deviations | Simple | Number of Protocol Deviations * Weight |
|  | *Data Quality* | Visits Entered within 7 Days | Percent-Based | (Number of Visits Entered within 7 days of occurring / Total Entered Visits) *Weight |
|  |  | Queries Answered within 7 Days | Percent-Based | (Number of Visits Entered within 7 days of occurring / Total Entered Visits) *Weight |

**Threshold-Based Games:**

Scoring in a threshold-based game takes a different form than the points-based examples above.  Instead of harmonizing multiple metrics using metrics, many goals for a given scoring period are set, and sites are ranked according to how many of them are met.

Setting these thresholds is typically very intuitive, as they usually represent site performance goals outlined in Key Performance Indicators (KPIs). You might already have a goal, for example, that sites need to enroll 3.5 patients per month for the study to meet enrollment timelines, or that sites should answer data queries within 7 days of their creation. In a points-based game, a simple metric such as enrollment count will take the form of X points per Y, a threshold game such a metric usually takes the form “Score if N events occur within P period.” The example of query resolution could take the form of “X% of queries answered within 7 days at time of scoring”.

In addition, certain other types of metrics can become targets for inclusion in a threshold-based game, as opposed to a points-based one. Metrics that would frequently incur a loss of points, such as the number of protocol deviations that occur are a prime example of this. Given that some number of protocol deviations are expected to occur, it is unwise to penalize a site per deviation. A site might even be well justified in committing major protocol deviations if they are in the best medical interest of the patient. In a threshold model, you can instead set a goal that allows for wiggle on such metrics while still aiming to minimize their incidence. A threshold in this area could look like, “Score if fewer than N deviations per patient are present at time of scoring.”

**Supplement Materials Table 6:** Scoring method of a threshold formatted version of the example Tour De France game. In this version of the game, the game is scored and presented monthly rather than scored live via a calculated dashboard.

| **Tour De France Metric** | **Threshold Monthly Goal** |
| --- | --- |
| Screening | 16 Screened Patients |
| Randomization | 2 Randomizations |
| Data Entry | 80% of Outcomes Visits entered within 48 hours of the visit |
| Protocol Deviations | Fewer than 1 major deviation per randomized patient |
| Webinars | Monthly Webinar Attended |
